# Advances in Traumatic Brain Injury Biomarkers

**DOI:** 10.7759/cureus.23804

**Published:** 2022-04-04

**Authors:** Kengo Nishimura, Joacir G Cordeiro, Aminul I Ahmed, Shoji Yokobori, Shyam Gajavelli

**Affiliations:** 1 Neurological Surgery, University of Miami, Miami, USA; 2 Wolfson Centre for Age-Related Diseases, King’s College London, London, GBR; 3 Department of Emergency and Critical Care Medicine, Division of Neurosurgical Emergency, Nippon Medical School, Tokyo, JPN; 4 Neuroscience, Lacerta Therapeutics, Alachua, USA

**Keywords:** brain trauma indicator, uchl-1, gfap, neurologic prognosis, serum biomarkers, traumatic brain injury, prognostic biomarkers

## Abstract

Traumatic brain injury (TBI) is increasingly a major cause of disability across the globe. The current methods of diagnosis are inadequate at classifying patients and prognosis. TBI is a diagnostic and therapeutic challenge. There is no Food and Drug Administration (FDA)-approved treatment for TBI yet. It took about 16 years of preclinical research to develop accurate and objective diagnostic measures for TBI. Two brain-specific protein biomarkers, namely, ubiquitin C-terminal hydrolase-L1 and glial fibrillary acidic protein, have been extensively characterized. Recently, the two biomarkers were approved by the FDA as the first blood-based biomarker, Brain Trauma Indicator™ (BTI™), via the Breakthrough Devices Program. This scoping review presents (i) TBI diagnosis challenges, (ii) the process behind the FDA approval of biomarkers, and (iii) known unknowns in TBI biomarker biology. The current lag in TBI incidence and hospitalization can be reduced if digital biomarkers such as hard fall detection are standardized and used as a mechanism to alert paramedics to an unresponsive trauma patient.

## Introduction and background

Overview of neurotrauma and traumatic brain injury diagnosis

Traumatic brain injury (TBI) is a burgeoning public health problem worldwide [1,2]. The US Department of Defense formally defined TBI as a “traumatically induced structural injury or physiological disruption of brain function as a result of an external force” [3], and interagency initiatives help widespread acceptance of the definition [4]. Upon a neurological examination, the following alterations would be evident: (i) any period of loss of or decreased consciousness; (ii) loss of memory for events immediately before or after injury (amnesia); (iii) focal neurological deficits such as muscle weakness, loss of vision, and change in speech; and (iv) alteration in mental state such as disorientation, slowed thinking, and confusion [4]. Within the United States, TBI accounts for over 300,000 hospital admissions and 2.2 million emergency department visits each year [5-7]. TBI is a form of acquired brain injury and is best diagnosed at the time of injury or within the first 24 hours. It may be open (penetrating) or closed (non-penetrating). The Glasgow Coma Scale (GCS) uses impairment of consciousness, a clinical hallmark of acute brain injury, as a diagnostic measure [8]. The GCS has provided a practical method for bedside assessment to categorize patients as mild, moderate, and severe [9]. Although less than 10% of TBI cases are severe, they account for more than 80% of the total related global cost which is estimated to be 400 billion dollars/year [10,11]. The management of severe TBI (featuring compromised cranial vault/space-occupying lesions, medically refractory intracranial hypertension) starts with surgical debridement, and despite its effectiveness in increasing survival, there is no clear correlation with improving outcomes [12,13]. TBI survivors are typically managed in the intensive care unit (ICU) setting, involving invasive monitoring to measure intracranial pressure (ICP), partial pressure of oxygen in brain tissue (PbtO_2_), and, less commonly, intracerebral flow, intracortical electroencephalogram (EEG), and microdialysis [14]. Trauma-related alterations to hemodynamics are fueled by prognostic biomarkers, coagulopathy, and cytokines such as interleukin-6 (IL-6) [15]. Coagulopathy measures such as activated partial thromboplastin time (aPTT), fibrin degradation product (D-dimer), fibrinogen, proteolytic product cleaved (C-tau) [16,17] predict long-term outcomes [18,19]. Despite maximal therapy, a large subset of patients never achieves a reasonable neurologic function. Conventional computed tomography (CT) scan in the acute phase is the cornerstone of TBI diagnosis. CT unequivocally detects lesions eligible for surgical intervention. Regardless of the GCS score after a certain threshold, an acute subdural hematoma is surgically evacuated, and in such cases “time is brain,” with the prognosis improving if a patient undergoes surgery less than four hours from trauma [20]. GCS scores of 13 to 14 are categorized as mild traumatic brain injury (mTBI). mTBI is survivable and does not need hospitalization in some cases but may impact productivity [21,22]. The mTBI diagnosis is often misunderstood. Subgroups of patients are at risk of developing delayed intracranial injury (ICI) that can be catastrophic, with 25% of such patients left severely impaired or dead [23-25]. Clinical decision rules based on the Scandinavian Neurotrauma Committee (SNC), Canadian CT Head Rule (CCHR), and New Orleans Criteria (NOC) developed evidence-based guidelines to identify patients at high risk for ICI. Only recently a “Transforming Research and Clinical Knowledge in Traumatic Brain Injury” (TRACK-TBI) study, externally validated by “Collaborative European NeuroTrauma Effectiveness Research in Traumatic Brain Injury” (CENTER-TBI), highlighted that contusion, subarachnoid hemorrhage, and/or subdural hematoma features were associated with incomplete recovery but not epidural hematoma [26]. A normal CT does not preclude brain injury and is of limited prognostic value [27,28]. The currently accepted definition of mTBI was established in 1993 by the American Congress of Rehabilitation Medicine (ACRM). Recently, a survey of an international, interdisciplinary group of clinician-scientists with expertise in mTBI identified several potential revisions to consider when updating the ACRM mTBI definition [29]. The ability to detect those patients could allow concentrating resources among the patients with chances of meaningful functional recovery. Hence, studies have been exploring alternative objective means to augment TBI diagnosis with blood tests, special brain imaging, eye movements, brain wave patterns, and portable imaging devices. This article presents the Food and Drug Administration (FDA) approval of the first blood-based biomarker test for the evaluation of mTBI in adults, along with possible ways to integrate digital biomarkers.

The need for this review was evident after the publication of our work on TBI patient samples [30] which captured some of the earliest perturbations after injury. However, despite numerous articles on TBI, biomarkers, and preclinical data, there were none describing (i) TBI diagnosis challenges, (ii) the process behind the approval of biomarkers, especially for neurotrauma, and (iii) known unknowns in TBI biomarker biology. Hence, for this foundational/scoping review, relevant recommendations listed by the Preferred Reporting Items for Systematic reviews and Meta-Analyses extension for Scoping Reviews (PRISMA-ScR) [31] were implemented. This was because at the time of manuscript submission there was only one FDA-approved TBI biomarker. Authors performed an electronic search of NCBI-PubMed, published between 1974 and 2022, and US FDA websites in English. The search terms included “Traumatic brain injury, Biomarkers, Neurotrauma diagnosis” excluding any references related to research in rodents and coronavirus disease 2019. Of the 277 citations, publications matching the biomarker glossary listed by the National Institutes of Health (NIH)-FDA workgroup were included.

## Review

Biomarkers

The 21st Century Cures Act Section 507 specifies the process of biomarker qualification. Biomarkers are measures that can help characterize a baseline state, a disease process, or a response to treatment [32]. The US FDA and the NIH published the first version of the glossary included in the Biomarkers, EndpointS, and other Tools (BEST) resource [32]. The goal is to harmonize and clarify terms used in translational science and medical product development and to provide a common language for communication. Application aids future discussions, trial planning, and clinical decision-making. These include a proper intent of use, discovery tools, sample collection, data collection, data analysis, assay development, assay validation, clinical performance, regulatory approval, and market access evaluation before a diagnostic test can be commercialized and used clinically. Following biomarker discovery, this evaluation process would first include analytical validation to assess the accuracy and reliability of the proposed test to measure the candidate biomarker. They can be molecular, histologic, radiographic, and/or physiologic types. Biomarkers are divided into seven categories.

**Figure 1:**
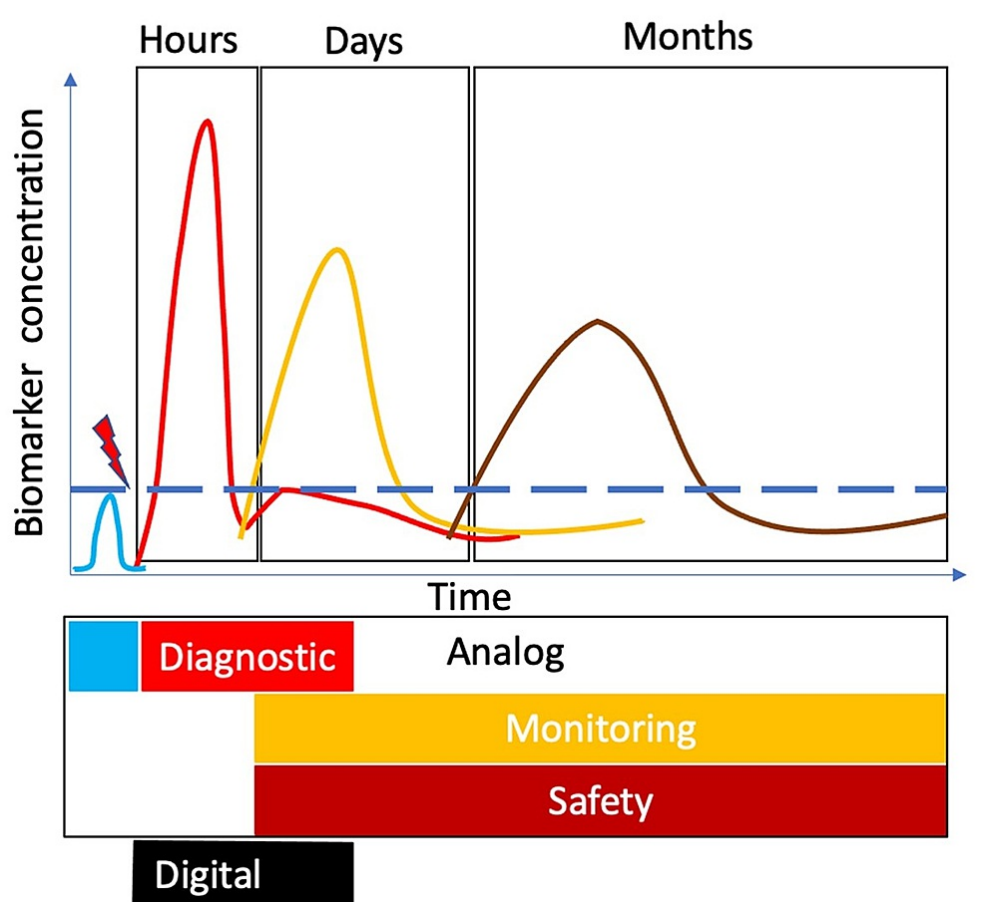
Specific biomarker types and their spatiotemporal distribution. TBI-specific biomarker types and their spatiotemporal distribution. The prevalence of a biomarker in a healthy population (blue) shifts following injury (red thunderbolt) to a new distribution (red) above the baseline (dashed horizontal line) that is not present in 99 percentiles of the uninjured (specific to injury). Such a biomarker can peak acutely (first rectangle), function as a diagnostic biomarker, and in absence of therapy fuel a cascade of secondary injury over days and months (next to rectangles). The subsequent subacute and chronic biomarkers (yellow and brown, respectively) could serve as monitoring/therapeutic response biomarkers. Failure of therapeutic effect can be assessed by increased safety biomarker. These analog biomarkers (black outline) can be augmented with digital biomarkers (black rectangle) to detect injury and alter, especially when an individual is alone and unresponsive following TBI. It is important to reinforce that any biomarker data should be interpreted only in the context of pertinent clinical history. TBI: traumatic brain injury

Each of these categories lends itself to different uses. A diagnostic biomarker can aid proper binary classification of subjects, for instance, an emergency department (ED) visitor seeking care into subgroups (outpatient versus inpatient) using an unbiased measure. The classifier boundary between subgroups must be determined by a threshold value of the measured object. The threshold can be assessed by comparing the prevalence of the biomarker in subgroups (Figure 1).

The four possible outcomes are as follows: true positive (sensitivity), true negative (specificity), false positive (type I error), and false negative (type II error). After establishing a pre-specified cutoff for the biomarker, discrimination can be assessed by the area under the receiver operating characteristic curve (AUC) with 95% confidence intervals (CIs). The deduced positive predictive value (PPV, the probability that subjects with a positive screening test truly have the disease) and negative predictive value (NPV, the probability that subjects with a negative screening test truly do not have the disease) help assess diagnostic accuracy [33]. Before such use in the clinic, preclinical studies are required to establish the following: (i) the association of the biomarker with TBI, (ii) correlation (linear relationship) with the presence of biomarker and injury severity and prognosis, (iii) capturing evidence to attribute TBI as the most likely/sole source of the biomarker with additional causalities such as retrodictive and predictive [34]. Using such data, a requestor can approach the FDA for approval of a TBI blood biomarker through a specific program, “The Breakthrough Devices Program,” part of the 21st Century Cures Act (Cures Act). This is a voluntary program for certain medical devices and device-led combination products that provide for more effective treatment or diagnosis of life-threatening or irreversibly debilitating diseases or conditions [35].

Biomarkers in Neurotrauma Diagnosis

In the years before CT, neurosurgical and diagnostic interventions were often based on recognizing changes in clinical signs and symptoms. To confirm a diagnosis, the patient was subjected to a risky, invasive angiogram with a tendency to wait too long as the patient was in extremis. Consequently, TBI was associated with high mortality rates. The existing Coma Index evolved into the Glasgow Coma Scale (GCS), which allowed earlier detection of a deteriorating patient [9,36]. TBI treatments are time-sensitive and resource-intensive [37]. An estimated 4.8 million ED visits per year are related to TBI [38-40]. The volume of work poses a substantial burden to the medical system, and this increase has been driven by a better understanding of mTBI along with interest in detecting concussion (representing a subset of mTBI without structural brain injury) early. Accurate clinical diagnosis of mTBI remains a challenge, and CT is not easily available (for instance, on the battlefield or on the pitch-side), is expensive, and imposes radiation exposure risk [41]. Hence, there is an unmet need to rapidly diagnose and manage mTBI more cost-effectively and efficiently without compromising patient care and safety. A sensitive blood-based marker that can be easily and rapidly measured in the systemic circulation would alleviate the burden imposed by the inability to diagnose TBI. Identification of biomarker signatures associated with distinct aspects of TBI pathophysiology may also be of clinical value for increasingly accurate characterization and risk stratification of TBI patients, optimizing medical decision-making, and facilitating individualized and targeted therapeutic interventions. TBI biomarkers can be distributed over the various phases post-injury, namely, acute, sub-acute, and chronic, capturing the initial damage, escalation, and progressive tissue loss [42]. Preclinical research has established the correlation and causality of TBI with induced biomarkers [43-47]. The application of proteomics in TBI research [48] accelerated the pace of TBI biomarker discovery, development, and validation. In this pioneering study, pooled naive and injured cortical samples (48-hour post-injury; male rat-controlled cortical impact model) were processed and analyzed using a differential neuroproteomics platform. Differential proteins were identified, quantitated, and confirmed. The differential proteins were categorized as: (i) decreased, (ii) increased, or (iii) putatively degraded by proteolysis. The neuronal protein ubiquitin carboxyl-terminal hydrolase-L1 (UCH-L1) was one of the proteins that increased in abundance. The preclinical study was followed by a clinical investigation of pediatric brain injury. Higher serum levels of two proteins, glial fibrillary acidic protein (GFAP) and UCH-L1, were detected in brain-injured children compared with controls. A step-wise increase in biomarker concentrations over the continuum of severity from mild to severe TBI was detected. Of the two, only the neuronal biomarker UCH-L1 had the potential to detect acute intracranial lesions, as assessed by CT. Most importantly, both markers were substantially increased in TBI patients with a normal CT. Serum UCH-L1 and GFAP concentrations also strongly predicted poor outcomes [49]. This led to the development and subsequent authorization by the US FDA of The Brain Trauma Indicator™ (BTI™), the first blood test to evaluate concussion in adults [50]. An overview of UCH-L1 and GFAP biomarkers measured by BTI™ in the blood within 12 hours post-injury is presented by Wang et al., and the turnaround time for the test is three to four hours [51]. This makes it a candidate for use in the ED or acute care setting. Levels of such biomarkers in the blood after mTBI can predict the presence of intracranial lesions in patients visible on CT scans. The TRACK-TBI study with a large prospective, multicenter, observational trial including adults (≥18 years) with head CT (part of standard emergency care) and blood collected within 12 hours of injury and prespecified cutoff values for UCH-L1 and GFAP (327 pg/mL and 22 pg/mL, respectively) reported that serum samples of 1,288 (66%) patients had a positive test result and 671 (34%) had a negative test result. The test had a sensitivity of 0.976 (95% CI = 0.931-0.995) and an NPV of 0.996 (95% CI = 0.987-0.999). In three (<1%) of the 1,959 patients, the CT scan was positive when the test was negative [52]. Recently, plasma GFAP measurements showed a high discriminative ability to predict intracranial abnormalities on CT in TBI patients across GCS 3-15 through 24-hour post-injury as a point-of-care (POC) platform prototype assay. GFAP substantially outperformed another serum biomarker, S100b [53]. Abbott Diagnostics’ prototype i-STAT POC version is touted to return results in 15 minutes after the sample is placed in the cartridge, such rapid turnaround may help transferability to the clinical practice [54]. Yue et al. demonstrated that the prototype i-STAT-device determined plasma levels of GFAP within 24 hours post-injury and could discriminate between magnetic resonance imaging (MRI)-positive patients and MRI-negative patients with an area under the receiver operating characteristic (ROC) curve of 0.777 [95% CI = 0.726-0.829). Abbott Diagnostics is now partnering with the US Department of Defense and the TRACK-TBI consortium to conduct a multicenter pivotal clinical trial on the i-STAT POC version of UCH-L1/GFAP tandem plasma tests for mTBI [51]. TBI serum biomarkers were demonstrated to be of greater value over clinical characteristics in predicting CT abnormalities, with correlations to clinical severity, and care path. The study examined six serum biomarkers including GFAP. Samples were obtained <24 hours post-injury from 2,867 patients with any severity of TBI in the Collaborative European NeuroTrauma Effectiveness Research (CENTER-TBI) Core Study, a prospective, multicenter, cohort study. GFAP achieved the highest discrimination for predicting CT abnormalities (AUC = 0.89; 95% CI = 0.87-0.90), with a 99% likelihood of being better than clinical characteristics. Results were consistent across strata, and injury severity supported a need to develop serum GFAP assays [55]. While the findings are being independently reproduced in studies with appropriate sample size [2], and commercialization of the test is in progress, polytrauma along with mTBI poses challenges that can probably be resolved by (i) rigorous standardization of the ACRM definition of mTBI [29], and (ii) harmonizing the testing methods to reduce inter-instrument variations [56,57].

Improving TBI diagnosis with new types of biomarkers

Analog Biomarkers

The aforementioned biomarkers can be considered analog (as opposed to digital) as the test is spatiotemporally separated from the time and place of biomarker incidence in the body. The BTI™ test is run on a semi-automated enzyme-linked immunosorbent assay (ELISA) platform that requires skilled technical personnel to operate and takes three to four hours to run while i-STAT POC could cut that time in half. Following the establishment of GFAP as the most discriminatory mTBI biomarker, molecules specific to astrocyte metabolism that are also associated with TBI could be excellent candidates. One such example is D-serine, which is a unique chirality D-handed amino acid that in physiological conditions is released by neurons to activate NMDA receptors, along with glutamate, which is important for synaptic transmission and plasticity [58,59]. Following TBI, there is a change from a phase release by neurons to a tonic glial release, which results in the hyperactivation of NMDA receptors and subsequent synaptic damage [60]. Another important anomalous phenomenon observed in TBI is dependence receptor-mediated cell death. Dependence receptors are defined as receptors that convert to pro-apoptotic death receptors following cellular stress and in the absence of ligand binding (e.g., EphB3) [30]. An extensive and thorough discussion raised important biological “known unknowns” that affect the reliability, reproducibility, and accuracy of blood-based protein biomarkers. These include (i) existence behind the blood-brain barrier, limitations imposed by the molecular size, direction of flow, and concentration gradients, half-lives in various biological fluid compartments; (ii) effect of preanalytical and analytical variables such as time, quality of sample collection, processing, and storage; and (iii) specificity and sensitivity of antibodies and existing analytical platforms [61]. In the case of proteins biomarker, digital ELISA-based multi-analyte assays could increase the sensitivity sufficient to detect a single molecule of the biomarker [42,62-64]. Similarly, nucleic acid biomarkers for TBI [65,66] detection can be improved via the adoption of the nucleic acid detection platform CRISPR-ENHANCE [67]. These approaches would complement the previously mentioned need to harmonize instrumentation and mTBI diagnostic criteria, as is being explored for imaging biomarkers [68].

Digital Biomarkers

One of the shortcomings of current biomarker studies of mTBI is reporting values at a single, and varying, post-injury time-point [61]. The availability of digital devices provides an opportunity to explore and adopt digital biomarkers as objective quantifiable measures [69,70]. Although an experimental study of intentional falls from a wheelchair among able-bodied young adults using Apple Watch showed poor sensitivity for fall detection [71], numerous anecdotes of improved outcomes from hard falls due to detection by Apple Watch consistently make the news. To assess the utility of such devices, a clinical trials (NCT04304495) GAPcare II has been initiated. It is proposed to provide insights into the feasibility, acceptability, and usability of the Apple Watch, iPhone, and the RIFitTest app for fall detection in older adults who are at high risk of recurrent falls and worse outcomes [72]. People at risk for TBI or following a TBI could use digital devices throughout their life to generate real-world data. Wearables and apps-generated collection of high-density data can give new powerful insights to rapidly respond to an incident and modulate or modify disease. Making meaningful conclusions from such data is challenging and is best addressed by artificial intelligence-based methods [61].

Table 1 discusses seven different biomarkers along with their definitions.

**Table 1:** Types of biomarkers. TBI: traumatic brain injury

	Types of biomarkers	Definition	Example in TBI	Authors
1	Susceptibility/Risk biomarker	A biomarker that indicates the potential for developing a disease or medical condition in an individual who does not currently have clinically apparent disease or medical condition	Age, gender, occupation, everyday activities, concussion, traumatic accident involving cranial impact, apolipoprotein E polymorphism	Wang et al. (2018) [73]; Maiti et al. (2015) [74]
2	Diagnostic biomarker	A biomarker used to detect or confirm the presence of a disease or condition of interest or to identify individuals with a subtype of disease	Glasgow Coma Scale, ubiquitin C-terminal hydrolase-L1, glial fibrillary acidic protein, computed tomographic scanning, magnetic resonance imaging	Wang et al. (2021) [51]
3	Monitoring biomarker	A biomarker measured repeatedly for assessing the status of a disease or medical condition or for evidence of exposure to (or effect of) a medical product or an environmental agent	Cerebral metabolic rates of brain oxygen, glucose, lactate/pyruvate ratio, arterial pressure, hypotension	Toro et al. (2022) [75]
4	Prognostic biomarker	A biomarker used to identify the likelihood of a clinical event, disease recurrence, or progression in patients who have the disease or medical condition of interest	Glasgow Outcome Score, Glasgow Outcome Scale-Extended, Disability Rating Scale, blood/serum-based biomarkers	McCrea et al. (2021) [76]; Wang et al. (2018) [73]
5	Predictive biomarker	A predictive biomarker is used to identify individuals who are more likely to respond to exposure to a particular medical product or environmental agent. The response could be a symptomatic benefit, improved survival, or an adverse effect	Cerebral metabolic rates of brain oxygen. Serum biomarkers	Gan et al. (2019) [77]
6	Pharmacodynamic/Response biomarker	A biomarker used to show that a biological response has occurred in an individual who has been exposed to a medical product or an environmental agent	None	Poloyac et al. (2019) [78]
7	Safety biomarker	A biomarker measured before or after an exposure to a medical product or an environmental agent to indicate likelihood, presence, or extent of toxicity as an adverse effect	Blood, plasma, serum biomarkers, e.g., plasma neurofilament light chain, thromboelastography	Scott et al. (2017) [79]

## Conclusions

Currently, BTI™ is the only FDA-approved biomarker in neurotrauma diagnosis. Abundant intracellular UCH-L1 and GFAP are present in neurons and astrocytes, respectively, in the brain parenchyma and are thought to be released into the body fluids by TBI. However, how they are subsequently metabolized remains unknown. Addressing such knowledge gaps in biomarker biology, standardizing mTBI inclusion criteria, and increasing detection sensitivity and assay speed can improve diagnostic accuracy which can be used for proper classification of patients. Combining the aforementioned analog biomarkers with modern digital assay platforms can shorten the interval between injury and admission to hospital. The advent of wearables will allow us to go beyond the snapshot of biomarkers in the clinic and facilitate continuous monitoring of populations at higher risk for TBI such that timely personalized intervention can be administered to prevent secondary injury (post-TBI seizures) and improve outcomes.
